# Genetic and Phenotypic Characterization of a *Pseudomonas aeruginosa* Population with High Frequency of Genomic Islands

**DOI:** 10.1371/journal.pone.0037459

**Published:** 2012-05-25

**Authors:** Rosario Morales-Espinosa, Gloria Soberón-Chávez, Gabriela Delgado-Sapién, Luisa Sandner-Miranda, José L. Méndez, Gerardo González-Valencia, Alejandro Cravioto

**Affiliations:** 1 Departamento de Microbiología y Parasitología, Facultad de Medicina, Universidad Nacional Autónoma de México, Mexico City, Mexico; 2 Departamento de Biología Molecular y Biotecnología, Instituto de Investigaciones Biomédicas, Universidad Nacional Autónoma de México, Mexico City, Mexico; 3 Unit of Medical Investigation in Infectious and Parasitological Diseases, Centro Médico Nacional, Siglo XXI, Instituto Mexicano del Seguro Social, Mexico City, Mexico; 4 International Center for Diarrhoeal Disease Research, Bangladesh, Dhaka, Bangladesh; University of Hyderabad, India

## Abstract

Various genomic islands, PAPI-1, PAPI-2, PAGI-1, PAGI-2, PAGI-3, and PAGI-4, and the element pKLC102 have been characterized in different *P. aeruginosa* strains from diverse habitats and geographical locations. Chromosomal DNA macroarray of 100 *P. aeruginosa* strains isolated from 85 unrelated patients hospitalized in an intensive care unit was created to assess the occurrence of these genomic islands (GEIs). The macroarray was then hybridized with labeled probes derived from each genomic island. In addition, PFGE patterns with *Spe*I, frequency of virulence genes, and antimicrobial resistance patterns of the strains were studied. Our results showed that almost all *P. aeruginosa* strains presented up to eight virulence genes. By *Spe*I macrorestriction fragment analysis we were able to identify 49 restriction patterns; 35 patterns correspond to single strains and the remaining 14 to strains subgroup (a–n). Most of the strains showed variation in number or composition of GEIs and a specific antimicrobial pattern indicating that each strain was an unrelated isolate. In terms of the number of genomic islands per strain, 7 GEIs were found in 34% of the strains, 6 in 18%, 5 in 12%, 4 in 14%, 3 in 10%, 2 in 7%, and 1 in 4%; only one isolate did not present any GEI. The genomic islands PAPI-1 and PAPI-2 and the element pKLC102 were the most frequently detected. The analysis of the location of each GEI in the chromosome of two strains show that the islands PAGI-3, PAPI-1, PAPI-2 and pKLC102 are present in the insertion site previously reported, but that PAGI-2 and PAGI-4 are inserted in another chromosome place in a site not characterized yet. In conclusion our data show that *P. aeruginosa* strains exhibited an epidemic population structure with horizontal transfer of DNA resulting in a high frequency of GEIs.

## Introduction

*Pseudomonas aeruginosa* is a gram-negative rod bacterium, which is reported to be ubiquitous in the natural environment, humans, and animals, and is an important opportunistic human pathogen that causes severe infections in immunocompromised patients [1]–[4]. It has been commonly associated with repeated or persistent bronchial infections in patients suffering from cystic fibrosis (CF), and it is a major cause of nosocomial infections, mainly in intensive-care units [5]–[7]. Many nosocomial infections are difficult to eradicate due to a number of factors, the most important of which is the relatively poor efficacy of antibiotics against *P. aeruginosa* due to multiple resistance mechanisms expressed by the bacterium [1], [2]. Several cell-associated and secreted virulence factors related to the bacterium have been described, which are encoded on plasmids or chromosomal genes, such as *lasB* (encoding for elastase), *toxA* (exotoxin-A), *pilA* (type fimbrial precursor type IV pilin), *plcH* (hemolytic phospholipase C precursor), *phzA1* (phenazine biosynthesis protein), *toxR* (transcriptional regulator), and *lecA* (lectin) [8]–[14]. Its ability to thrive in a broad range of environments is partially due to a large and diverse genome [12], [15]–[21]. The bacterium presents a picture of a mosaic genome consisting of a conserved core component interrupted in each strain by combinations of specific blocks of genes. These strain-specific segments of the genome are found in limited chromosomal locations, referred to as genomic islands (GEIs), which are acquired by horizontal gene transfer (HGT). Depending on the functions they encode and the advantage they confer relative to the specific lifestyle of a bacterium, GEIs can be called pathogenicity, symbiosis, fitness, metabolic, or resistance islands [22]–[25]. Furthermore, the presence of identical genes in the pathogenic and non-pathogenic variants of one species – for example, in extraintestinal pathogenic and commensal *Escherichia coli* – implies that some of these encoded functions contribute to general adaptability, fitness and competitiveness, rather than to particular virulence traits [26]. A large number of GEIs in the *P. aeruginosa* chromosome have been described; however, these GEIs are found in variable numbers in some strains and not in others [27]. Studies performed to date have identified and characterized several islands. The genomic island PAGI-1 was first identified in a urinary tract infection isolate, the sequence analysis of which revealed a length of 48,893-bp with 51 predicted open reading frames (ORFs), and present in 85% of the studied clinical strains [28]. The islands PAGI-2 and PAGI-3 were discovered in the strains C and SG17M respectively; PAGI-2 has a length of 104,955-bp with 111ORFs, while PAGI-3 contains a portion of strain-specific DNA sequence of 103,304-bp with 106 ORFs. In both strains, C and SG17M, the genomic islands are partitioned into two blocks. The cluster adjacent to the *attL* site consists of genes that are specific to each strain, while the other cluster predominantly contains hypothetical ORFs of which 47 are mutual homologs in both genomic islands [29]. *Pseudomonas aeruginosa* genomic islands PAPI-1 and PAPI-2 have been identified in the genome of PA14, a highly virulent clinical isolate [15]. The PAPI-1 island has a size of 107,899-bp with 115 predicted ORFs and has a highly mosaic structure. Remarkably, more than 80% of its DNA sequence is unique and shows no similarity to any GenBank sequences. Conversely, the other ORFs-translated sequence show homology to proteins from several bacterial species. Significantly, many PAPI-1 ORFs also occur in several P. aeruginosa cystic fibrosis isolates, and approximately 11 genes are required for virulence in plants and animals [15], [30], [31]. PAPI-2 occupies a DNA region of 10,722-bp and an organization of 15 predicted ORFs, half of which encode to hypothetical proteins of unknown function [15]. pKLC102 is a 103,532-bp integrative and conjugative element initially found in the *P. aeruginosa* clone C strain SG17M that can exist as a plasmid or integrate into the chromosome, and can excise from the chromosome at a rate of up to 10%. This element revealed 105 coding sequences (CDS), 60 of which were classified as hypothetical or of unknown origin. Many of these hypothetical genes have DNA replication, recombination, and modification genes as neighbors. Syntenic sets of homologous genes were identified in other plasmids and genomic islands among gram-negative bacteria, including PAGI-2 and PAGI-3 of *P. aeruginosa* clone C strains [32], [33]. The island PAGI-4 has a length of 23.4-kb and is integrated at the 3′ end of the tRNALys gene of the strain C. The 9.5-kb segment adjacent to the tRNALys gene is homologous not only with sequences of the chromosomal and episomal versions of pKLC102 in strain C but also with the tRNAGly-associated genomic island PAGI-2 [32]. Seven novel genomic islands have been identified, PAGI-5 -6, -7, -8, -9, -10 and -11, with sizes varying from 99 to 2-kb and containing a total of 201 ORFs among them. Several are related to known pathogenicity islands, phages, or *Rhs* elements while others are quite novel [34], [35].

The aim of this study is the phenotypic and genetic characterization of a large collection of *Pseudomonas aeruginosa* strains, isolated mostly from patients with pneumonia at an intensive-care unit (ICU) of a specialty hospital from Mexico City, analyzing the frequency of several GEIs (PAPI-1, PAPI-2, PAGI-1, PAGI-2, PAGI-3 and PAGI-4), an integrative element pKLC102 and of different virulence factors. To our knowledge, this is the first report of the high prevalence in a single ICU of *P. aeruginosa* strains with multiple GEIs.

## Materials and Methods

### Bacterial Strains

A collection of 100 clinical strains of *P. aeruginosa* were used in this study. The clinical strains were isolated between January 2005 and November 2006 from 85 patients admitted to the intensive care unit (ICU) of Hospital de Especialidades at Centro Medico Nacional, Siglo XXI in Mexico City. 7 of 85 patients presented 2 different strain isolates on different dates, while other 4 patients were infected with 3 different strains. Most of the patients were diagnosed with ventilator-associated pneumonia, with the remaining cases being diagnosed with septicemia and meningitis. The clinical strains were obtained from different sources: 60 were isolated from bronchial washings, 18 from sputum, 10 from blood, 2 from throat swabs, 2 from cerebrospinal fluid, 1 from pleural fluid, and 7 from unknown origin. The reference strains used as positive controls were *P. aeruginosa* PAO14 and *P. aeruginosa* clone C strains: C and SG17M, which were provided by Dr. B. Tümmler from Hannover, Germany. *P. aeruginosa* PAO1 was used as a negative control. All the strains were maintained in 15% glycerol at −70°C. Each strain was biochemically typed using conventional biochemical tests and the API20 NE system. All strains showed biochemical patterns of *P. aeruginosa*. The project was approved by the Ethics Committee of the Hospital de Especialidades of the Centro Medico Nacional, Instituto Mexicano del Seguro Social, and CIE (32-2007, Comisiones de Investigación y Ética) Medicine School Universidad Nacional Autónoma de México. Mexico City, Mexico. In all cases patients or relatives were informed about the nature of the study and were asked to sign a consent form.

### Virulence Genes Detection

Seven structural and virulence *P. aeruginosa* genes (*toxA*, *lasB*, *lecA*, *algR*, *plcH*, *phzA*1 and *toxR*) were selected and amplified by PCR with specific primers designed in the laboratory, the annealing temperature of each primer pair is shown in Table 1. For *pilA* gene detection, we designed a probe derived from the conserved region corresponding to 5′ end of *pilA* gene, it is located from nucleotides 5234035 to 5233992 with respect to strain UCBPP-PA14 and from nucleotides 5069556 to 5069473 with respect to the strain PAO1 (Table 1). Additionally, knowing the variability of *pilA* gene, we completed its detection using a primer set proposed by Kus *et* *al*. [36], which was designed to amplify from the conserved *pilB* gene to the tRNA^Thr^ gene.

**Table 1:** Primer sequence used in the genetic characterization of *P. aeruginosa* clinical strains.

Gene/GEIs*	Forward primer sequence (5′–3′)	Reverse primer sequence (5′–3′)	Tm	Size of PCR product	Reference
Virulence gene					
*toxA*	TCAGGGCGCACGAGAGCAACGAGA	GACAGCCGCGCCGCCAGGTAGAGG	66.1°C	454pb	This study
*lasB*	ACTGTCGCGGCCGCATTTCGTCAT	CATCGCCGTGCCGTCCCAGTAGG	65°C	433pb	This study
*lecA*	CGATGTCATTACCATCGTCG	TGATTGCACCCTGGACATTA	65°C	215pb	This study
*algR*	AGGGCAACTGGACGGCTATC	TGTGGTCGGCAATGAAGAAGA	63°C	437pb	This study
*plcH*	CGACGAGGGCGACGGCTTCTATGA	CCGGGCAGGCTCTTGGGCTCGTA	66°C	447pb	This study
*phzA1*	AACCACTTCTGGGTCGAGTG	GTGGGAATACCGTCACGTTT	65°C	203pb	This study
*toxR*	ATGGCATCTATGCGAGGAAC	GCAGGGGAATGAAGTTCTTG	65°C	207pb	This study
*pilA*					
*pilB/tRNAThr* **^Φ^**	TCCAGCAGCATCTTGTTGACGAA	CGAATGAGCTGCTCTACCGACAGAGCT	55°C	1–4-kb	Kus, 2004
*pilA* probe	TGATCGAACTGATGATCGTGGTTGCGATCATCGG		58°C	-	This study
*oprL* **^Φ^**	AGGGCGGCGATGCTTCC	CGACGCGACGGTTCTGAG	61.1°C	420pb	This study
*lipA* **^Φ^**	CAAGCCGGGCAAGGTGGAAGTCG	CGGATCTCGCGCAGGCAGTCG	65.1°C	456pb	This study
**PAGI-1**					
*orf3*	TGGTGCTGACCAGCGACAAG	TCCATCGACTCGGTGCGTAG	60°C	958pb	Finnan, 2004
*orf18*	ATTCCTCCACTGCCGTTCACAACG	CCTTGCTCATCTGGAACAGGTAGC	60°C	1039pb	Finnan, 2004
*orf42*	CGGAGAACCATCTCTCGCACAC	GGCTAAGACGTTCGACTGATTCC	60°C	675pb	Finnan, 2004
**PAGI-2**					
*c22*	CCTTCGTCCATTACCTGTGGAAC	AACTTGCGAGCCAACTCACG	62.4°C	943pb	Finnan, 2004
*c105*	GATTGATGCTCAACGACGATGG	GCTGTTCCGCCTTCAGTTCC	59°C	681pb	Finnan, 2004
**PAGI-3**					
*sg8*	TACAGAGTGCCCGAGCTGATG	GTGCTTCCCTGAGAGACAGACG	62°C	732pb	Finnan, 2004
*sg100*	GCAATCTGTACGTCCTGCACG	AGCACGGCTTGTCGCTGTTC	62°C	553pb	Finnan, 2004
**PAGI-4**					
*CL22*	CATGATCCGGCACACTGAGGTC	ATGATGGCGAGCGCTACAAGGTTC	60.6°C	464pb	This study
**PAPI-1**					
976F/PAPI-1R	GCCTGACGGTGTCCTGTTAT	GCTGCCTCTCCTACGAACA	58°C	2600pb	Qiu 2006
4542F/intF	GTGGTGATGACCTCCAACCT	AGCTACATCGAGGCCGACTA	58°C	1600pb	Qiu 2006
SojR/4541F	CGAGCACAGAAATGTCCTGA	GACAAGACCAGCCACAACCT	58°C	1600pb	Qiu 2006
IntF/sojR	AGCTACATCGAGGCCGACTA	CGAGCACAGAAATGTCCTGA	58°C	1600pb	Qiu 2006
**pKLC102**					
*cp10*	CGGACCACTAGATAGCCAGG	GGACGCCATTGAGTATGCGC	61°C	255pb	Klockgether 2007
*cp44*	GGGTCCGCAAAACTTTCCGC	GCTTGAGGTTGGGCCAATCG	61°C	272pb	Klockgether 2007
*cp97*	GGATATCTACGTACCCCGGC	CTTTTTACCCGCAGTGGCGG	61°C	337pb	Klockgether 2007
**PAPI-2**					
*xerC* PAPI-2 1F/1R	TGTTCCGCTCGGGTGCCTTCATC	CACGCATCACTCCCGCCTGGTTC	66°C	417pb	This study
RS07-RS08 PAPI-2 2F/2R	GGCGAGGTCCAGAATGTGTCAGG	TCCCCGCCCGCAGAGTCA	66°C	402pb	This study
*exoU* PAPI-2 3F/3R	GCGGCGCAACGACAACCTGAT	GAAAAGCCACCGCCCCGTCTGT	66°C	434pb	This study

* Genomic islands, Φ Marker gene for hypervariable region location.

### Probes Used for GEIs Detection

We amplified by PCR the specific genes ORF3, ORF18 and ORF42 of PAGI-1; C22 and C105 of PAGI-2; and SG8 and SG100 of PAGI-3; using the primer pairs described by Finnan [37]. We amplified genes CP10, CP44 and CP97 of pLKC102 using the primer pairs described by Klockgether [33]. The selected genes represent the left portion, right portion, and middle region of each island. We used the primer sets proposed by Qiu [31] to amplify different DNA segments of PAPI-1: the primer pair 976F and PAPI-1R detected the integration of PAPI-1 at the *attB* site in the locus PA0976; the primer pairs 4542F + intF and sojR + 4541F were used to amplify and detect the left and right junction sequences between the chromosome and island respectively when it is integrated into the chromosome at the *attB* site in the tRNA^Lys^ gene locus PA4541.1 corresponding to the strain PA14, and finally the primer pair intF + sojR was used to detect the presence of a circular PAPI-1 (Table 1). We designed in the laboratory three pairs of primers to amplify three specific regions of PAPI-2: the primers PAPI-2-1F and PAPI-2-1R were used to amplify the *xerC* gene localized at locus RS02 at the left region of PAPI-2; the primers PAPI-2-2F and PAPI-2-2R were used to amplify the middle region (locus RS07 and RS08) of the island, and the primers PAPI-2-3F and PAPI-2-3R were used to amplify the *exoU* gene (locus RS14) localized at the right region of the PAPI-2. We designed a primer set to amplify the gene CL22, this gene was chosen because it is a specific gene of PAGI-4 without significant similarity with other gene reported in the databases (Table 1). PCR protocols to amplify each gene belonging to PAGI-1, PAGI-2, PAGI-3, pKLC102 and PAPI-1 were done according to authors' instructions [31], [33], [37]. Thermal cycling condition for the genes of PAPI-2 and PAGI-4 were: an initial denaturation cycle at 94°C for 2 min, followed by 35 cycles at 94°C for 1 min, annealing temperature (according to each specific primer set [Table 1]) for 1 min and 72°C for 1 min with a final cycle of 72°C for 2 min. All PCR products of each gene were used as probes, which were labeled with DIG High Prime DNA labeling according to the manufacturer's instructions (Roche Applied Science, Germany).

### Chromosomal DNA Isolation and Macroarray printing

Chromosomal DNA was isolated from overnight cultures of each of the 100 clinical *P. aeruginosa* strains analyzed in this work, as well as the 4 *P. aeruginosa* reference strains, (PA14, PAO1, C and SG17M). DNA was purified from bacteria by miniprep (DNeasy Blood & Tissue Kit QIAGEN) as previously described, and adjusted to 100 ng/μl. An aliquot of 40 μl of each DNA was dispensed individually into a 384-well plate, denatured for 30 mins at 65°C, and chilled on ice. Each DNA was spotted by duplication onto nylon membranes by Virtek's ChipWriter System robot. Macroarray copies were produced in parallel from the same stock of DNA to ensure that the corresponding spot was represented by identical amounts of sample on each membrane.

### Hybridization of Macroarray

Membranes were incubated for 3 hrs at 60°C with hybridization buffer (Roche Diagnostics), and hybridized overnight at 60°C using the same buffer with Dig-labeled PCR product (corresponding to each selected gene). The membranes were washed twice for 30 min at 65°C in washing buffer (0.1 M maleic acid, 0.15 M NaCl, pH 7.5, 0.3% (v/v) Tween 20). Detection of DIG-labeled fragments and exposure to X-ray film were performed according to manufacturer's instructions. A presence/absence determination was made by comparison with hybridization signals obtained with reference strains.

### Macrorestriction Analysis

Genomic DNA in agarose blocks was prepared using the method previously described by Liu [38] with some modifications. Briefly, bacteria were grown overnight (for no more than 15 hrs.) in 5 ml of Luria-Bertani (LB) broth and harvested by centrifugation. The bacterial pellet was washed in 500 µl of cool PIV (10 mM Tris [pH 8], 1 M NaCl). This procedure was repeated up to 5 times, depending on the amount of alginates present in the culture and adjusted to 5 OD at 600 nm. Then, 200 µl of the cell suspension was mixed with an equal volume of 1.5% low melting temperature agarose. The mixture was drawn into disposable plug molds (BioRad) and cooled at 4°C. The agarose plugs were incubated overnight at 37°C in 2 ml of lysis solution (1 M Tris [pH 8], 1 M NaCl, 0.5 M EDTA [pH 8], 0.5% sodium deoxicolate, 12.5% N-lauroyl-sarcosine, 5µg/ml RNAse, 10 µg/ml lysozyme), then the blocks were incubated again overnight at 50°C in ESP (10 mM Tris HCl [pH 7.4], 1 mM EDTA, 0.25% N-lauroyl-sarcosine, 0.1 mg/ml proteinase K [Sigma]). A second incubation in ESP solution was performed in order to increase the purity of the DNA. Finally, blocks were washed 7 times (for 1 hr. each) with cool TE solution (Tris-HCl 10 mM [pH 8], 1mM EDTA [pH 8]) and stocked in TE buffer at 4°C. Agarose blocks were pre-incubated in 1X buffer used for *Spe*I enzyme for 30 mins at 37°C and then incubated with 100 µl of fresh 1X buffer containing 30 U of *Spe*I at 37°C overnight to digest DNA in the blocks. *Spe*I fragments were separated by a CHEF-DR II device (Bio-Rad) and electrophoresis was performed on 1.2% agarose gels and 0.5X TBE buffer at 10°C with pulse time ramped from 5 to 25 s over 19 hrs and 5.3 V/cm and a second block with pulse time ramped from 5 to 60 s over 17 hrs and 5.3 V/cm. Sizes of *Spe*I fragments were estimated using *Xba*I fragments of *Salmonella braenderup* global standard H9812. Gels were stained with ethidium bromide, photographed and then analyzed using whole band analyzer software (Bioimage). To determine similarity of *Spe*I fingerprints, a presence/absence band matrix was constructed and then a dendrogram was generated by the Neighbor-joining method using Nei's minimum distance (1972). The algorithms were implemented in Tools for Population Genetic Analyses software [39]. Clustering of profiles was determined under the criterion of >80% of band match (Biolmage-Whole Band Analyzer).

### Location of the GEIs within the Hypervariable Regions in the Strains Chromosome

Six strains with different chromosomal restriction profiles and with 7 GEIs in their chromosome were selected: strains 12, 58 and 99 from subgroup “a”, and strains 124, 127 and 128 from subgroup “n”. The location of the 7 GEIs and of the hypervariable regions on the chromosome of each strain was carried out by Southern blot hybridization on the *SpeI* macrorestriction profile, using as specific probes the ^32^P labelled PCR products of the marker genes of each GEI and from the genes *lipA*, *oprl* and *pilA* for the detection of each hypervariable region (Megaprime DNA labeling system, [Amersham Biosciences]). The membranes were incubated in 10 ml of hybridization solution (Rapid-hyb buffer) to which 1X Denhardt's solution and 0.2mg/ml of denatured sheared salmon sperm were added and incubated at 60°C with constant and gentle shaking for 3 h. The labeled probe was then added to fresh hybridization solution and hybridization was carried out overnight at 60°C with constant and gentle shaking. After being washed at high astringency (64°C) the membrane was exposed to a X-ray film. The location of each GEI was deduced from the comparison of its positive hybridization with the positive hybridization of each hypervariable region.

### Antimicrobial Susceptibility

To assess the susceptibility profiles to 20 antimicrobial agents of the 100 strains of *Pseudomonas aeruginosa* isolated from Mexican adults with pneumonia, the agar dilution method was used according to the guidelines established by the National Committee for Clinical Laboratory Standards (NCCLS) [40]. ATCC 27853 *Pseudomonas aeruginosa*, ATCC 25922 *Escherichia coli, ATCC 35218 Escherichia coli, ATCC 29213 Staphylococcus aureus*, and *ATCC 29212 Enterococcus faecalis* were used as controls in the susceptibility tests. All the strains were grown in Muller Hinton agar and harvested in sterile saline solution to achieve a turbidity equivalent to that of a No. 0.5 McFarland opacity standard. The antimicrobial agents tested against *P. aeruginosa* were: carbenicillin (16–64 µg/mL), ticarcillin (8–32 µg/mL), piperacillin (1–8 µg/mL) ticarcillin/clavulanic acid (8/2–32/2 µg/mL), piperacillin/tazobactam (1/4–8/4 µg/mL) ceftazidime (1–4 µg/mL) ceftriaxone (8–64 µg/mL) cefotaxime (8–32 µg/mL) cefepime (1–8 µg/mLl) imipenem (1–4 µg/mL) meropenem (0.25–1 µg/mlL) aztreonam (2–8 µg/mL) amikacin (1–4 µg/mL) gentamicin (0.5–2 µg/mL) tobramycin (0.25–1 µg/mL) polymyxin b (0.25–2 µg/mL) ciprofloxacin (0.25–1 µg/mL) norfloxacin (1–4 µg/mL) and levofloxacin (0.5–4 µg/mL). Agar dilution was performed using two-fold increments (across a range of 0.125 to 512 μg/mL) of each antimicrobial agent incorporated into Muller-Hinton agar. The concentration range for susceptibility and resistance are indicated in parenthesis with a MIC value lower than the cut-off to indicate susceptibility and two-fold dilutions above the cut-off to determine resistance. The criterion for intermediate susceptibility was based on isolates growing within one-fold dilution higher than the MIC value.

### Statistical Analysis

Linkage disequilibrium to measure the amount of recombination within the population sampled was determined using the standardized index of association I_A_ [41]. The mean genetic diversity H was calculated using the same software.

## Results

### Frequency of Virulence Genes

PCR was used to assess the prevalence of eight virulence genes of which *toxA, toxR, algR*, *lasB, lecA, plcH*, and *phzA1* were detected in 100% of the strains, while *pilA* gene was detected by hybridization only in 55% of isolates, but its detection increased to 98% when PCR primers were used to amplify complete *pilA* locus. Only two strains were *pilA* negative.

### Frequency of GEIs

The results show that in 99% of the strains at least one genomic island was present. Of the 100 strains tested, 34% presented all the GEIs: PAGI-I, PAGI-2, PAGI-3, PAGI-4, PAPI-1 and PAPI-2 and the studied insertion element pKLC102; 18% presented 6 GEIs of which the majority were PAGI-2, PAGI-3, PAGI-4, PAPI-1, PAPI-2 and pKLC102; 12% strains presented 5 GEIs of which PAPI-2, PAGI-2, PAPI-1, pKLC102 were always present, in combination with PAGI-4 or PAGI-3; 14% presented 4 GEIs of which PAGI-2 and pKLC102 were always detected; 10% of the isolates presented 3 GEIs, PAPI-2 and pKLC102 in combination with PAGI-4 or PAGI-2; 7% presented the genomic island PAGI-2 and pKLC102 element; and 4% of the strains presented only one GEI. There was only one strain that did not present any GEI. Finally, the island PAPI-2 and the element pKLC102 were the most frequently detected among the strains (87%) followed by PAPI-1 (81%), PAGI-2 (78%), PAGI-4 (70%), PAGI-3 (53%) and PAGI-1 (51%).

**Figure 1:**
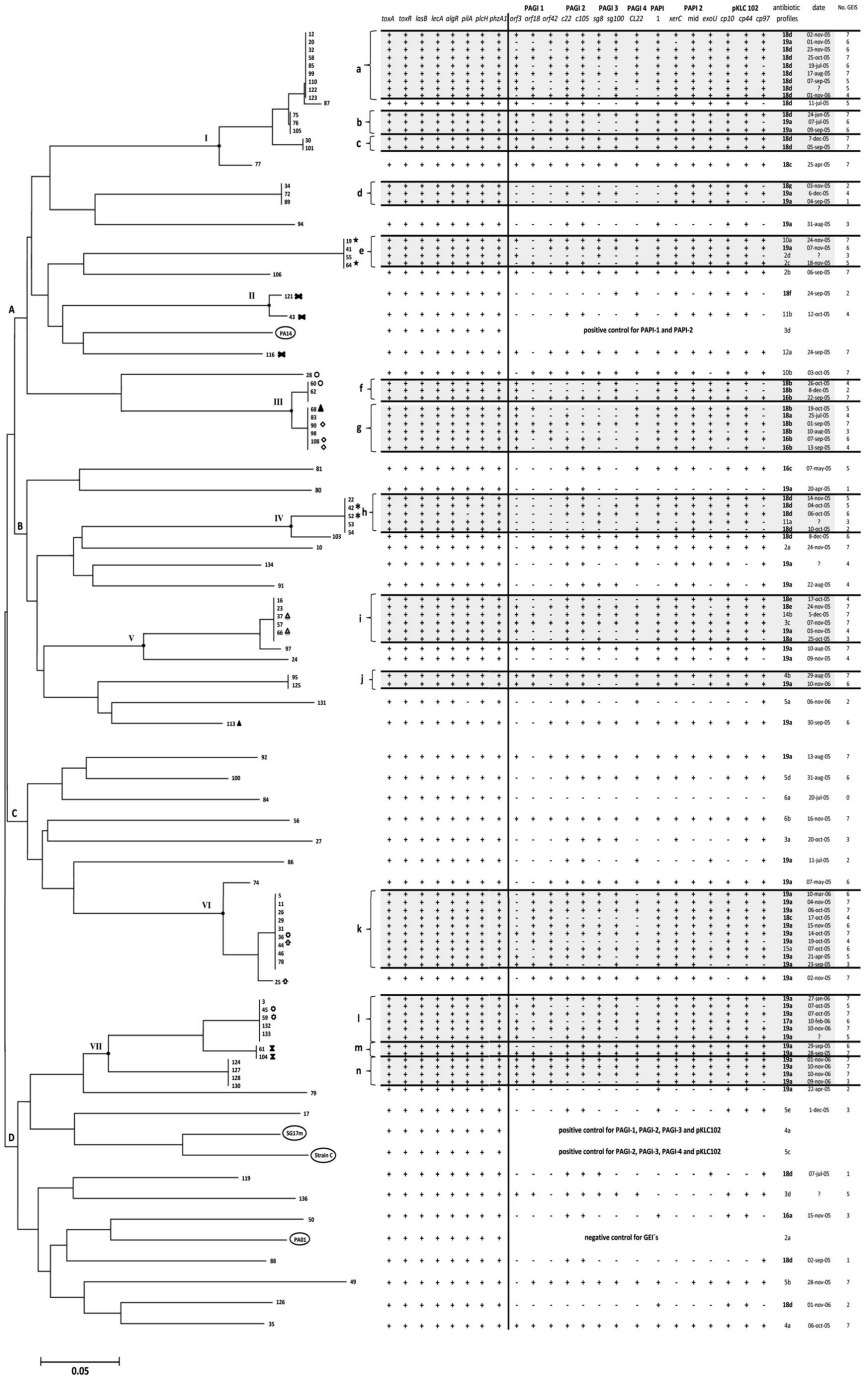
Unrooted dendrogram for 100 *Pseudomonas aeruginosa* clinical isolates. The Neighbor-Joining dendrogram was constructed using PFGE restriction patterns (*Spe* I) based on a distance generated matrix (Nei's [1972] Minimum Distance). Upper case letters (A–D) represent the four generated clades. Clustering of profiles shown in roman numerals was determined under the criterion of >80% of band match (BioImage-Whole Band Analyzer). Low case letters (a–n) represent strain subgroups. Presence (+) of virulence genes were determined by PCR and presence (+) of GEIs by hybridization with specific labeled probes. Antibiotic resistance profiles were performed by agar dilution method according to NCCLS. Symbols beside the strain number indicate isolates from the same patient.

Additionally, the detection of each marker gene of the genomic islands was variable among strains, showing diversity in their genetic content. For example, in the detection of PAGI-1, hybridization was positive for ORF3 in 49 strains, for ORF18 in 43 strains, while ORF42 was positive in 50 strains; 31 strains showed positive hybridization with two of the three ORFs and less than half of the strains (20) showed positive hybridization with the three ORFs (Figure 1). In the detection of PAGI-2, hybridization of gene C22 was positive in 80 strains and of C105 was positive in 79 strains, with 78 strains being positive for both genes. With regard to PAGI-3 detection, hybridization was positive with the probe derived from gene SG8 in 59 strains, while 61 strains were positive for SG100 and 53 strains for both genes. In the detection of pKLC102, there was also variability, gene CP10 was positive in 87 strains, CP44 was positive in 88 and CP97 in 70 strains, more than half of the strains (63) presented all three genes. PAPI-1 was found to be integrated into the chromosome at locus PA0976.1 in 65% of the isolates, and at locus PA4541.1 in 16% of the isolates. The circular form of the genomic island was not detected in any of the strains included in this study.

### Macrorrestriction Analysis

**Figure 2:**
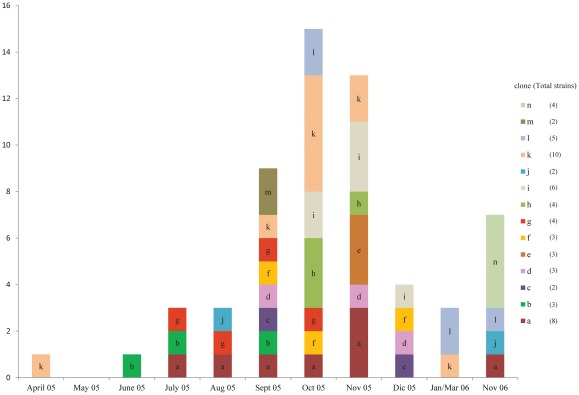
Histogram showing the prevalence of strains genotype (a –**n) over time (Apr 05–Nov 06).** The genotype of the *Pseudomonas aeruginosa* strains was obtained by *Spe*I macrorestriction fingerprint.

According to *SpeI* fragment patterns, the strains were distributed in four major clades (A–D), seven clusters (I–VII), and several individual patterns (Figure 1). Reference strains PAO1, C, and SG17M were grouped in clade C showing each individual restriction pattern, while strain PA14 was grouped in clade A. In terms of genotyping by *SpeI* fingerprint, we were able to identify 49 genotypes, represented for 49 different restriction pattern of which 35 corresponded to 35 single strains (unique patterns), and the 14 remaining to 14 subgroups (a–n). With respect to the prevalence of each strain, we found no relationship between the presence of a strain with a particular chromosomal profile type and its date of isolation. Each strain appeared in different months, but neither of them remained present for more than 5 consecutive months nor through all the period of the study (Figure 2). For example, the strains of subgroup “k” appeared for the first time in April of 2005 and for last time in March of 2006, although these strains were only isolated in three different and non-continuous months. The strains of subgroup “J” appeared in August and were isolated again in November, 2006. The strains of subgroups “a” and “g” were present during five and four continuous months, respectively (Figure 2); and only one strain of subgroup “a” was isolated 12 months later. The strains of subgroups “e”, “m” and “n” were detected for a single month without further detection. Although, each subgroup grouped a determined number of strains with the same chromosomal profile, the majority of the strains were isolated from unrelated patients and most of them showed a variable number of GEIs and/or a different antimicrobial resistance profiles. Additionally, among the strains with the same number of GEIs and from the same subgroup differences among the types of GEIs and/or its marker genes were detected. Finally, the subgroups “e”, “g”, “h”, “i”, “k”, “l” and “m” grouped up to three strains isolated from the same patient at different times, and although the strains isolated from a same patient presented the same *Spe*I fingerprint each of them also had a variable number of GEIs with different antimicrobial resistance profiles, indicating that they were unrelated isolates (Figure 1). In this study, the distribution of *P. aeruginosa* variants revealed that there was not a prevalent strain colonizing our patient population.

### Location of the GEIs within the Hypervariable Regions in the Strains Chromosome

**Figure 3:**
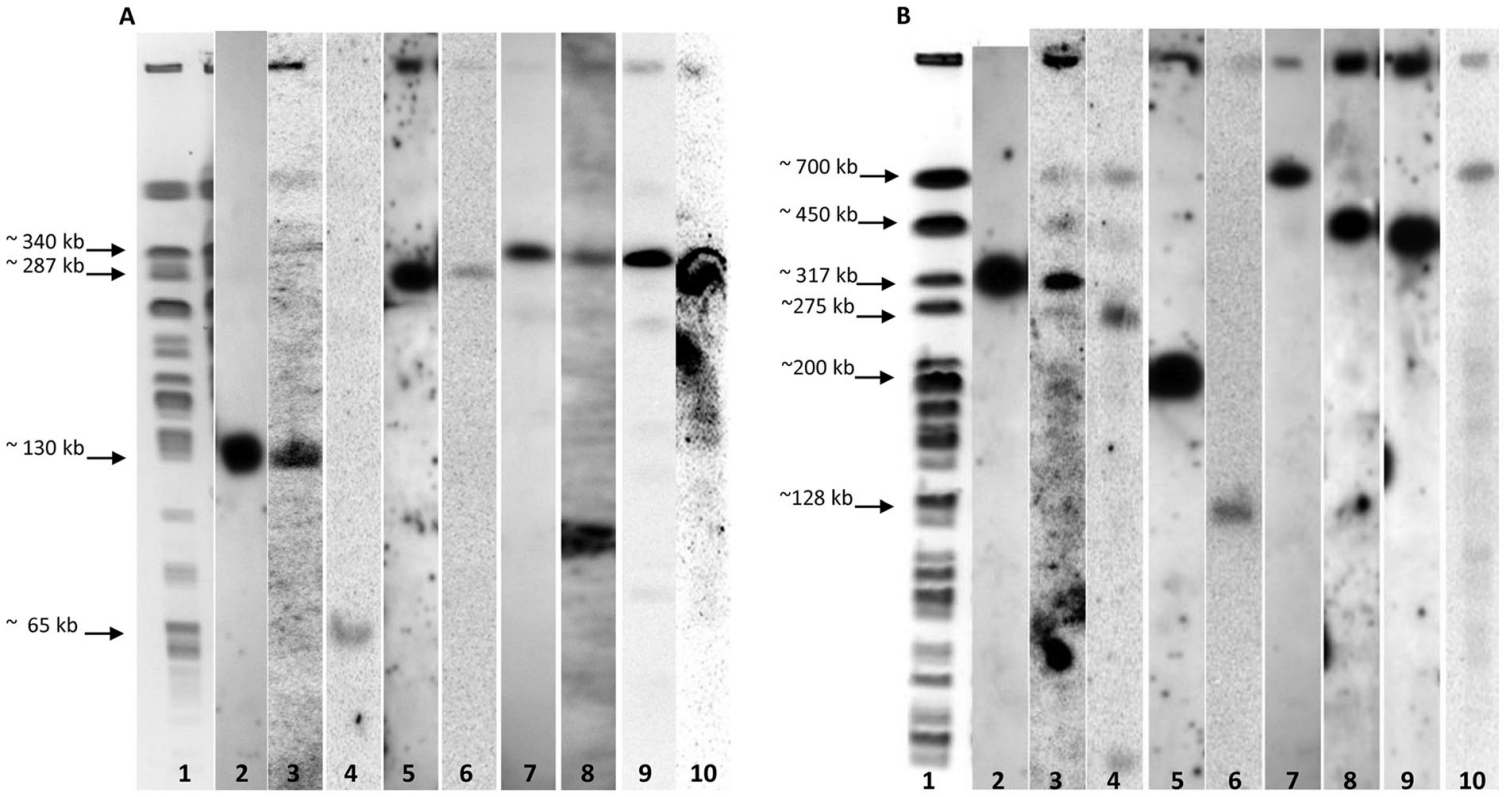
Location of hypervariable regions and genomic islands on *SpeI* chromosomal map of two *Pseudomonas aeruginosa* strains. (A) Lane 1 shows the chromosomal restriction pattern with *Spe*I of the strain 12 from subgroup “a”. Lane 2 shows the location of the hypervariable region close to *lipH* locus obtained for positive hybridization on a band of 130-kb with the marker gene *lipA*. Lane 3 shows the position of PAGI-3 on the band of 130-kb of chromosomal restriction pattern. Lane 4 shows the position of PAGI-2 on a band of 65-kb contrary to the expected size of 130-kb. Lane 5 shows the location of the hypervariable region close to *oprL-phnAB* loci obtained for positive hybridization on a band of 287-kb with the marker gene *oprL*. Lane 6 shows the position of PAPI-2 on the band of 287-kb of chromosomal restriction pattern. Lane 7 shows the position of PAGI-4 on a band of 340-kb of chromosomal restriction pattern contrary to the expected size of 287-kb. Lane 8 shows the location of the hypervariable region close to *pilA* locus obtained for positive hybridization on a band of 335-kb with the marker gene *pilA*. Lane 9 shows the position of pKLC102 on the band of 335-kb of chromosomal restriction pattern. Lane 10 shows the position of PAGI-1 with a positive hybridization on a band of 284-kb of chromosomal restriction pattern. (B) Lane 1 shows the chromosomal restriction pattern with *Spe*I of the strain 127 from subgroup “n”. Lane 2 shows the location of the hypervariable region close to *lipH* locus obtained for positive hybridization on a band of 317-kb with the marker gene *lipA*. Lane 3 shows the position of PAGI-3 on the band of 317-kb of chromosomal restriction pattern. Lane 4 shows the position of PAGI-2 on a band of 275-kb of chromosomal restriction pattern, contrary to the expected size of 317-kb. Lane 5 shows the location of the hypervariable region close to *oprL-phnAB* loci obtained for positive hybridization on a band of 200-kb with the marker gene *oprL*. Lane 6 shows the position of PAPI-2 on a band of approximately 130-kb of chromosomal restriction pattern. Lane 7 shows the position of PAGI-4 on a band of 700-kb of chromosomal restriction pattern, contrary to expected size of 200-kb. Lane 8 shows the location of the hypervariable region close to *pilA* locus obtained on a band of 448-kb with the marker gene *pilA*. Lane 9 shows the position of pKLC102 on the band of 448-kb of chromosomal restriction pattern. Lane 10 shows the position of PAGI-1 on a band of 706-kb of chromosomal restriction pattern.

The hypervariable regions adjacent to *lipH, oprL-phnAB* and *pilA* loci in the chromosome of the strains of subgroup “a” were located by southern hybridization in a *Spe*I restriction map. These regions hybridized in bands of 131-kb, 287-kb and 335-kb respectively, and in the strains of subgroup “n” in the bands of 706-kb, 200-kb and 448-kb respectively (Figure 3). The genomic islands PAGI-3 and PAPI-2, and the element pKLC102 hybridized in the bands corresponding to the marker genes (*lipA*, *oprL* and *pilA* respectively) of the hypervariable regions indicating that they are inserted within the regions reported by the authors. The islands PAGI-2 and PAGI-4 hybridized in bands of different size (Figure 3) indicating that these islands are in other chromosomal position different to *lipH* and *oprL-phnAB*, for example: the strains 12, 58 and 99 of subgroup “a” were positive in the band of 65-kb for PAGI-2 and in the bands of 340-kb, 256-kb and 287-kb for PAGI-4; the strains of subgroup “n” were positive in the band of 275-kb for PAGI-2 and in the band of 700-kb for PAGI-4. With respect to the location of PAPI-1, the insertion of this island has been already detected at the tRNA^Lys^ gene (PA0976.1) of the hypervariable region close to *oprL-phnAB* when we obtained the PCR product amplified with primers set PAPI -1 and 976F. Finally, the island PAGI-1 hybridized in the strains of subgroup “a” in two bands of different size of 284-kb and 205-kb; and in the strains of the subgroup “n” the island was located in the band of 706-kb size.

### Association Index and Genetic Diversity

Statistical analysis of the PFGE data for the 49 genotypes revealed an association index (IA) value close to 0 (0.0024, p<0.05), indicating linkage equilibrium and that recombination is frequent enough to break up clonal formation [41]. However, when calculated on the basis of all isolates, IA increased to 0.022, which suggested an epidemic population structure with frequent recombination among members of the population and occasional emergence of clones that successfully spread and persist for a while within a limited geographic and temporal span [42]. The mean genetic diversity H was 0.2749 +/−0.0143, which is in accordance with that found in many human pathogens with narrow ecological specificity. Others authors have reported a mean genetic diversity of 0.229 [43] and 0.357 [44] for *P. aeruginosa*.

### Antimicrobial Susceptibility Profile

**Figure 4:**
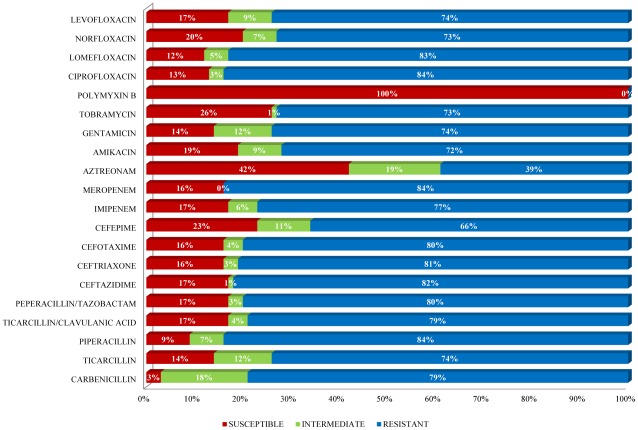
Histogram showing antimicrobial susceptibility profile of 100 *Pseudomonas aeruginosa* clinical strains to 20 common antimicrobials.

The determination of susceptibility profiles of 100 isolates to 20 antimicrobial agents showed 34 different resistance profiles, with 100% of strains showing susceptibility to polymyxin B, while the 73% of strains were resistant to 18 antimicrobials in different combinations. Only 15 strains were susceptible to a wide range of the antimicrobial tested (from 14 to 18). In general, the multi-resistance rate among our strains was very high (Figure 4).

## Discussion

Bacterial pathogenicity is evoked by the presence of multiple virulence factors encoded by groups of genes present in the chromosome and pathogenicity islands that interact in various combinations [11], [12], [45], [46]. *Pseudomonas aeruginosa* harbors several virulence genes used to colonize, destroy, and spread through tissue. Our results show that all the virulence genes are present in all strains of the present study and form part of the core genome of *P. aeruginosa*. These findings are in agreement with those studies that suggest that the different virulence genes are harbored by all *P. aeruginosa* strains independently from the sites of isolation, clinical, or environmental sample [14], [47].

The genome of a bacterial pathogen is composed of a conserved “core” genome, which contains the genetic information that is required for essential cellular functions, and a “flexible” gene pool, or accessory genome, that includes large insertions acquired by HGT that encode adaptive traits, which may be beneficial for bacteria under certain growth or environmental conditions. In *P. aeruginosa*, extensive genomic rearrangements have been reported, as well as acquisition or loss of large blocks of DNA contributing to genome size variations between 5.2 and 7Mb [17], [20]. In the *P. aeruginosa* chromosome there are three hypervariable regions [16], [17] that reside in the vicinity of the *lipH, phnAB* and *pilA* loci, where tRNA genes has been identified as the hot spots for the integration and excision of large DNA blocks [15], [28], [29], [31]–[33]. As it has been pointed out in previous studies, the insertions of the majority of the genomic islands take place within a specific region of the chromosome, and the strains that present one or two GEIs inserted in any of their hipervariable regions are few, suggesting that these GEIs are strain-specific and site-specific. However, contrary to those reports, our results show that one strain can harbor up to 7 GEIs in its chromosome and the frequency of each one of them is high in our *P. aeruginosa* population, suggesting that the functional competition and site-specific insertion among these genetic elements is negligible, probably because there are other sites of insertion in the chromosome that have not been characterized so far. Although, some authors have reported the frequency of some GEIs in their *P. aeruginosa* populations [33], [37], [48], there are few studies reporting the presence of several genomic islands in the chromosome of a single isolate of *P. aeruginosa* and unfortunately there are no other epidemiological data that shows the frequency from up to 7 islands in other *P. aeruginosa* populations with which the results from the current study can be compared.

Analysis of the insertion sites of each genomic island into the chromosome of the strains of the current study showed that PAGI-3, PAPI-1 and PAPI-2 and the element pKLC102 were located each in the hypervariable regions adjacent to *lipH, phnAB* and *pilA* respectively, as have been reported by the authors [15], [28], [29], [31]–[33]. We suppose PAPI-1 and PAPI-2 presented a tandem array located at tRNALys gene PA0976.1 (region *pilA*) at the same chromosomal location as in strain PA14. In two strains of subgroup “a” the genomic island PAGI-1 was located in the same band of PAPI-2 suggesting that in these strains there could be up to three genomic islands in the same DNA fragment. However, this event would not represent any problem, since it is reported that the island PAGI-1 is not inserted into any tRNA gene, so that the three islands could coexist without any competition for their sites of insertion. With respect to the islands PAGI-2 and PAGI-4, we found that their location in the chromosome is different from the reported, out of the hypervariable regions adjacent to *lipH* and *oprL-phnAB* respectively, indicating that there are other insertion sites in chromosome which have not been characterized yet. Sequencing of these DNA segments will need to be carried out to determine the specific site of insertion of PAGI-2 and PAGI-4.

In this study, the detection of each genomic island by hybridization with specific probes on a chromosomal DNA macroarray were successfully performed, allowing us to work with a large number of *Pseudomonas aeruginosa* strains. Additionally, the marker genes selected were adequate, showing high specificity and sensitivity for their detection. In a previous work Klockgether and coworkers [33] documented that PAGI-2 and pKLC102 were characterized in different subtypes according to their hybridization patterns, showing that these islands were variable in genes content when compared with the island hybridization patterns from one strain to another. In the present study, this difference could be seen in the hybridization patterns with the different marker genes used for the detection of PAGI-2 and pKLC102. Furthermore, these differences between the hybridization patterns of the marker genes were also seen among the islands PAGI-1, PAGI-3 and PAPI-2 suggesting that these islands also present different subtypes.

Little is known about the role of each genomic island in the virulence and adaptive traits of the bacteria. The majority of the proteins encoded within these islands have unknown functions and, in addition, they possess genes that encode functions related to DNA mobilization, integration, conjugation and partition activities [15], [31]. There are reports that try to associate the presence of some GEI with a particular pathology. It is known that PAGI-2 presents gene clusters encoding all nine essential proteins for the cytochrome c biogenesis system I and related thio-disulfide exchange protein. The expression of the genes for cytocrhrome c biogenesis could facilitate iron uptake and inactivation of peroxides [49], and thus, may confer an advantage for the bacteria to persist in the CF lung, where they are exposed to iron limitation and oxidative stress [50], [51]. The islands PAPI-1 and PAPI-2 have been associated in murine acute pneumonia and bacteremia infection models [52]. PAPI-1 carries several regulatory genes, including *pvrR*, which controls the biofilm formation of antibiotic resistant variants of *P. aeruginosa* that are associated with chronic infections in individuals with cystic fibrosis [15], [53]–[55]. However, to date the involvement of each island in the development of any particular disease is not clear. The high frequency of islands PAGI-2, PAPI-1 and PAPI-2 found in the majority of our strains isolated from lung secretions of patients with pneumonia, could be explained for their participation in the development of pneumonia as was pointed in previous studies.

However, what would be the participation of the remaining islands in this pathology? It is unknown, whether the presence of several GEIs interact synergistically. Probably the presence of all GEIs in the *P. aeruginosa* chromosome is to increase the pathogenicity of the strain, to favor multidrug resistance and/or to promote a better adaptation to the hospital and pulmonary environment. We do not know which is the frequency of these islands in strains isolated from other pathologies (urinary tract infection or septicemia), or in strains isolated from the natural environment in Mexico, but we know that the strains isolated from patients with pneumonia present a high frequency of GEIs.

The complete genetic and phenotypic characterization of 100 strains of *P. aeruginosa* associated with pneumonia infection in patients admitted to the intensive care unit of a highly specialized hospital in Mexico, showed that the infections were caused by unrelated strains with a great genomic diversity and that there was no cross-infection between patients associated with a single clone. The results support the idea that *P. aeruginosa* exhibits an epidemic population structure, which is predominantly sexual with the occasional emergence of clones that are only distributed and persist for a short time without causing outbreaks within the hospital environment.

We speculated that the high incidence of GEIs present in the collection of studied strains could be associated with multidrug-resistance present in these strains and the type of patients (hospitalized in ICU) from which the strains were isolated. However, this hypothesis cannot be proven yet since strains of *P. aeruginosa* isolated from other sources with low antimicrobial resistance profiles need to be studied.

### Final Conclusions

We can say that the high frequency of GEIs detection in *P. aeruginosa* strains in our population suggested: first, GEIs are not specific of strains and some are inserted elsewhere within chromosome in uncharacterized sites yet; second, the horizontal transfer of genes among our strains is common, leading to high contents of GEIs; and third, our strains are different to the strains and clones circulating in other parts of the world.
